# Comparing the CFIR-ERIC matching tool recommendations to real-world strategy effectiveness data: a mixed-methods study in the Veterans Health Administration

**DOI:** 10.1186/s13012-023-01307-x

**Published:** 2023-10-12

**Authors:** Vera Yakovchenko, Carolyn Lamorte, Matthew J. Chinman, David E. Goodrich, Sandra Gibson, Angela Park, Jasmohan S. Bajaj, Heather McCurdy, Timothy R. Morgan, Shari S. Rogal

**Affiliations:** 1 Center for Health Equity Research and Promotion, VA Pittsburgh Healthcare System, University Drive C (151C), Building 30, Pittsburgh, PA 15240-1001 USA; 2 Mental Health Research, Education, and Clinical Center, VA Pittsburgh Healthcare System, Pittsburgh, PA USA; 3 RAND Corporation, Pittsburgh, PA USA; 4 Division of Gastroenterology, Hepatology, and Nutrition, University of Pittsburgh, Pittsburgh, PA USA; 5 Department of Veterans Affairs, Office of Healthcare Transformation, Washington, DC, USA; 6 Division of Gastroenterology and Hepatology, Central Virginia VA Health Care System, Richmond, VA USA; 7 Division of Gastroenterology, Hepatology, and Nutrition, Virginia Commonwealth University, Richmond, VA USA; 8 VA Ann Arbor Healthcare System, Ann Arbor, MI USA; 9 Gastroenterology Section, VA Long Beach Healthcare System, Long Beach, CA USA; 10 Department of Medicine, University of California Irvine, Orange, CA USA; 11 Department of Surgery, University of Pittsburgh, Pittsburgh, PA USA

**Keywords:** Cirrhosis, Hepatocellular carcinoma, Implementation determinants, Implementation strategies

## Abstract

**Background:**

Practical and feasible methods for matching implementation strategies to diagnosed barriers of evidence-based interventions in real-world contexts are lacking. This evaluation compared actual implementation strategies applied with those recommended by an expert opinion-based tool to improve guideline-concordant cirrhosis care in a Veterans Health Administration national learning collaborative effort.

**Methods:**

This convergent parallel mixed-methods study aimed to (1) identify pre-implementation Consolidated Framework for Implementation Research (CFIR) barriers to cirrhosis care through focus groups with frontline providers, (2) generate 20 recommended strategies using focus group identified barriers entered into the CFIR-Expert Recommendations for Implementing Change (ERIC) Implementation Strategy Matching Tool, (3) survey providers over two consecutive years on the actual use of 73 ERIC strategies and determine strategy effectiveness, (4) compare actual versus recommended strategy use, and (5) compare actual versus expected barriers by reverse applying the CFIR-ERIC Matching Tool.

**Results:**

Eighteen semi-structured focus groups were conducted with 197 providers representing 95 VA sites to identify barriers to quality improvement, including cirrhosis care complexity, clarity of national goals, and local leadership support. The CFIR-ERIC Matching Tool recommended strategies such as assessing for readiness and needs, promoting adaptability, building local groups, preparing champions, and working with opinion leaders and early adopters. Subsequent strategy surveys found that sites used the top 20 “recommended” strategies no more frequently than other strategies. However, 14 (70%) of the top recommended strategies were significantly positively associated with cirrhosis care compared to 48% of actual strategies. Reverse CFIR-ERIC matching found that the strategies most used in the first year corresponded to the following barriers: opinion leaders, access to knowledge and information, and resources. The strategies most frequently employed in the second year addressed barriers such as champions, cosmopolitanism, readiness for implementation, relative priority, and patient needs and resources. Strategies used in both years were those that addressed adaptability, trialability, and compatibility.

**Conclusions:**

This study is among the first to empirically evaluate the relationship between CFIR-ERIC Matching Tool recommended strategies and actual strategy selection and effectiveness in the real world. We found closer connections between recommended strategies and strategy effectiveness compared to strategy frequency, suggesting validity of barrier identification, and application of the expert-informed tool.

Contributions to the literature

- The implementation science field must continue to refine barriers to strategy-matching methods and tools.
- This is one of the first studies to show that CFIR-ERIC Matching Tool recommended strategies, based on barriers collected qualitatively from local stakeholders, are associated with engaging in an evidence-based practice in a national effort.
- Our forward and reverse CFIR-ERIC Matching Tool approach could serve as a methodologic and analytical example for linking barriers to strategies.

## Background

The implementation science field has recognized that current methods for choosing implementation strategies that address diagnosed contextual barriers are either inherently subjective, not driven by empirical evidence, or frequently involve complex theory applications as to render them too complicated to replicate in applied implementation efforts [1–3]. While new tools [4–6] have been proposed to improve this process, no gold standard method exists to prospectively recommend implementation strategies. Researchers have developed an Excel-based matching tool [6], which allows users to input implementation barriers, defined by the Consolidated Framework for Implementation Research (CFIR), to receive recommendations about expert-recommended implementation strategies (ERIC) that may address these barriers. However, this tool is based solely on expert opinion. Therefore, there is a critical need to systematically evaluate how well this and similar tools function in the real world.

Ultimately, the goal is to provide frontline practitioners with theory-based guidance and validated tools that are efficient, reliable, and user-friendly to ensure improvement efforts result in the sustainment of the practice and the process changes impacting patient outcomes [7]. The Veterans Health Administration (VA) is a learning healthcare system which conducts rigorous evaluations of large-scale quality improvement (QI) initiatives [8, 9]. VA implementation scientists are often embedded within these large operational improvement initiatives to enable the health system to rapidly learn which strategies work best and under what pragmatic circumstances [10]. For example, the VA national Hepatic Innovation Team (HIT) learning collaborative spearheaded a system-wide effort to eliminate hepatitis C virus (HCV) among VA patients [11–13]. Using a survey we developed to assess ERIC strategy use, we previously reported the core implementation strategies employed by this collaborative as well as the individual VA sites nationwide delivering HCV treatment [14].

After tremendous success in treating nearly all Veterans with HCV between 2014 and 2018 [11, 15], the HIT Collaborative pivoted to focus on improving cirrhosis care given that only 33% of eligible VA patients receive all elements of guideline-concordant cirrhosis care [16–19]. Cirrhosis is the fourth leading cause of death among middle-aged adults in the USA, largely as a consequence of undertreatment of HCV and hepatitis B virus, increasing rates of obesity leading to non-alcoholic fatty liver disease, and alcohol-associated liver disease [19–21]. Cirrhosis requires complex multidisciplinary chronic disease management, including evaluation and treatment of complications and regular surveillance for hepatocellular carcinoma (HCC, i.e., liver cancer) [16]. Thus, barriers and strategies to cirrhosis care improvement were anticipated to be distinct from those needed to increase HCV treatment. The pivot was intended to learn about the unique barriers to cirrhosis care improvement and to address variations in care [22]. Given our ability to assess national use of implementation strategies over time, this was an ideal opportunity to compare the CFIR-ERIC Matching Tool recommendations to real-world strategy use and effectiveness data.

The aims of this evaluation were to (1) identify pre-implementation CFIR barriers to liver cancer surveillance through focus groups with frontline providers, (2) generate 20 recommended strategies using the CFIR-ERIC Implementation Strategy Matching Tool, (3) survey providers over two consecutive years on the actual use and effectiveness of 73 strategies, (4) compare actual versus recommended strategy use, and (5) compare actual versus expected barriers by reverse applying the CFIR-ERIC Matching Tool.

## Methods

### Design

This convergent parallel mixed-methods study identified and compared actual versus recommended implementation strategies and actual versus expected implementation barriers. Participants were clinicians, staff, and regional leaders from VA sites across the country. Participation was voluntary, and all findings were reported in aggregate to leadership at the VA HIV, hepatitis, and related condition program in the Office of Specialty Care Services with the goal of improving the HIT Collaborative program. Per regulations outlined in VA Program Guide 1200.21 [23], this project was deemed a non-research operation activity.

**Fig. 1:**
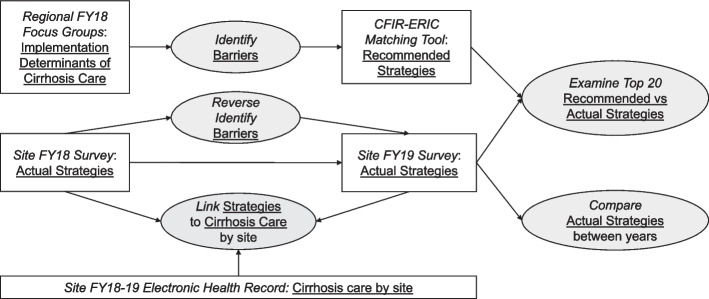
Study design. Note: VA fiscal year is October-September; CFIR: Consolidated Framework for Implementation Research; ERIC: Expert Recommendations for Implementing Change; Box: data collection; Oval: analysis, interpretation

Qualitative and quantitative data were collected from four primary data sources across two consecutive years. The fiscal year 2018 (October 2017 to September 2018) served as a “pivot year,” during which the HIT focused on pre-implementation for cirrhosis and sustainability for HCV while retaining the original HIT Collaborative infrastructure. The fiscal year 2019 (October 2018 to September 2019) was the second pivot year and the first complete year of cirrhosis care improvement implementation. Qualitative methods followed SRQR guidelines [24]. Figure 1 illustrates our concurrent qualitative and quantitative data collection and analytic process.

### Determinant framework

We structured our mixed-methods analyses according to CFIR [25], an implementation meta-framework that synthesizes and merges theoretical constructs from implementation frameworks and models to define potential factors that can influence the success of an implementation effort. CFIR includes 39 determinants that can positively or negatively influence implementation across five domains: *intervention characteristics*, *inner* and *outer settings* of the organization, *characteristics of individuals* involved in the implementation, and *implementation process*.

### Implementation strategies

We used the ERIC study’s compilation of 73 implementation strategy terms and definitions established by expert consensus to systematically track and evaluate activities [26]. The ERIC study proposed clustering the 73 specific strategies into nine thematic categories [27], ranging from *changing infrastructure* to *developing stakeholder interrelationships*, *supporting clinicians*, or *engaging patients*.

### Matching determinants to implementation strategies

Recent research has sought to optimize the selection of ERIC strategies based on conceptual linkages to CFIR barriers, resulting in the creation of the CFIR-ERIC Matching Tool. This expert-informed tool prioritizes which strategies are most closely associated with addressing negative CFIR determinants (i.e., barriers), thus producing a set of corrective implementation strategies based on barrier inputs. To arrive at these matchings, implementation science experts and practitioners (*n* = 169) used a ranking method to select and rank the “top 7” strategies that best address barriers related to CFIR barriers [6]. A table of 2847 (39 × 73) cells is generated, and a percentage is assigned to each cell based on the proportion of respondents endorsing the strategy for a specific barrier. The tool is an Excel macro worksheet with dichotomous barrier selection. In the case of multiple barriers input into the tool at once, endorsements are summed across rows to generate recommended strategy totals. The current functionality of the CFIR-ERIC Matching Tool does not consider enablers/facilitators; therefore, they were not explored in analysis. However, the future plans include expanding the tool to also include enablers.

### Data collection and analysis

#### Focus groups

The semi-structured interview guide was developed using the established CFIR interview guide [28], including questions from the five CFIR domains tailored to assess determinants related to improving cirrhosis care. For example, a question in the *Intervention Characteristics* domain was, “How complicated is it to improve HCC surveillance?”.

Focus groups were conducted virtually (Skype, telephone) with providers from each of 18 Veteran Integrated Service Networks (VISNs) between July and September 2018, leveraging existing VISN-level teams of providers working in the same geographical areas. Individual interviews were conducted when clinicians were not able to attend their regional focus group. All calls were audio recorded, and verbal informed consent was obtained from participants both prior to and at the start of recording. We recruited participants through the HIT Collaborative email distribution list and during a recurring monthly national HIT call. Participants (hereafter referred to as “providers”) included physicians, pharmacists, advanced practice providers (APPs), or other frontline roles, working in gastroenterology, infectious disease, pharmacy, quality improvement, and operations. Interviews lasted an average of 90 min. Live notes were taken to capture key quotes and responses for rapid memoing. NVivo, Excel, and Word were used for qualitative data management and analysis. Interviewers (VY, SSR, SG) represented different educational backgrounds (MPH, MD/MPH, BS), occupations (Health Science Specialist, Physician, Research Assistant), and levels of clinical knowledge and qualitative experience.

Qualitative coding and analysis were performed by two members of the evaluation team (CL, VY) using the Rigorous and Accelerated Data Reduction analysis technique [29]. Differences in coding were adjudicated by a third member (SSR). Responses were deductively themed to the five CFIR domains and their select constructs valence rated on a 3-point Likert scale as barrier (-1), facilitator (+ 1), or neutral (0) using established practices [30].

#### Survey of implementation strategies

As previously detailed, we tailored the 73 generic ERIC implementation strategies to dichotomous items (yes/no) on a 15-min survey focused on cirrhosis care improvement [31]. Surveys were emailed to “key informants” from each VA, who could report on cirrhosis care, asking them to reflect on the prior fiscal year (FY18 and FY19). The reported strategies were termed “actual” strategies and descriptively summarized for the pre-implementation year (FY18) and the first year of full cirrhosis QI implementation (FY19).

#### Barrier to strategy mapping tool

We used the CFIR-ERIC Matching Tool [6] to generate “recommended” implementation strategies based on barriers reported during focus groups. Each CFIR construct deemed a barrier was used in the Excel query. The resulting output includes the percent endorsement by individual barriers and the cumulative percentage endorsement denoting the most to least recommended strategies. The top 20 highest cumulative percentage strategies became the “recommended” strategies.

Next, we selected the top 20 strategies *used* in FY18 and FY19, based on the strategy surveys. We then compared the overlap of strategies between the recommended strategies to those that were used.

We also used the CFIR-ERIC Matching Tool in a reverse or backward manner to identify which barriers were being addressed by the strategies that were used in FY18 and FY19. For example, if the audit and provide feedback strategy was used then the most addressed CFIR barrier would be Goals and Feedback.

#### Cirrhosis care indicator

Strategies and barriers were anchored to FY18 and FY19 so they could be linked to cirrhosis quality measures over the same time period. Specifically, QI efforts focused on HCC surveillance among Veterans with cirrhosis. Using the VA Corporate Data Warehouse, all Veterans with cirrhosis (based on ICD-10 codes for cirrhosis or its complications) who had an encounter in VA in the prior 18 months and were not receiving hospice services were included in the denominator of Veterans with cirrhosis. Receipt of HCC surveillance was defined using radiology codes, health factors, encounter and inpatient procedure codes, and non-VA care fee-basis procedure codes [32]. Patient data were aggregated to the VA site (unit of analysis for strategy survey). Point-biserial tests assessed correlations between strategies and HCC performance to determine strategy effectiveness. The proportion of actual significant strategies (of 73) was then compared to the number of effective strategies within the recommended (of 20).

## Results

### Focus groups

#### Participants

A total of 197 HIT members from 95 unique sites (73%) and 18 regions (100%) participated in 18 focus groups (range 3–16 participants) and seven individual interviews. Participants were 75% female and included nurses (37%), APPs (22%), pharmacists (17%), physicians (14%), and system redesign or other frontline roles (10%), working in gastroenterology, infectious disease, pharmacy, and operations to improve liver disease care in VHA. Focus groups identified barriers to and facilitators of cirrhosis quality improvement efforts across the five CFIR domains during the first year of a national learning collaborative pivoting from HCV to cirrhosis care improvement.

### Implementation barriers

Barriers were primarily from the CFIR *inner setting*, *intervention characteristics*, and *outer setting* domains.

### Intervention characteristics

#### Complexity

Participants universally emphasized the complexity of cirrhosis care, particularly in contrast to HCV treatment (e.g., cirrhosis “*is a complete different animal, much more complex, and not that easy to implement*”). Where HCV can be identified using lab data, identifying patients with cirrhosis requires more clinical training and involves more provider discretion. Thus, many sites faced ongoing challenges with accurately identifying the true cohort of patients with cirrhosis.

Logistically, the complexity of managing recurring imaging studies for HCC surveillance was a central concern for participants and raised the issues of staff distribution, including the “need [for] admin support” to handle the complexity of “post-discharge scheduling, pulling patients in, linking to clinics, HCC screening, and ultrasound every six months [with] patient no-shows and a difficult patient population.”

#### Adaptability

In part because of the complexity of cirrhosis care, individual sites were perceived to have different needs, requiring adaptations to address the fact that, “each site will look different.” This was particularly true for sites without specialty care: “rural facilities don’t have hepatologists…[specialists] aren’t geographically located where patients need the care.” Participants thus recommended a focus on adapting and tailoring care processes for sites relying on community providers for cirrhosis care.

### Outer setting

#### Policies, incentives, and metrics

Participants described the need for clear performance metrics, goals, and national policies and directives promoting cirrhosis care. One clinician said few sites “are routinely engaged because no one knows exactly what the metrics are or how to use them—no agreed upon standard.” Another clinician explained, “until there is a national metric they are held to; it is hard to get it done—clear-cut direction will be helpful.”

#### Patient needs and resources

Engaging Veterans was noted as challenging because cirrhosis is often a silent disease. One participant lamented, “linkage to care is hard and many patients [are] asymptomatic, treated by primary care, and are under impression that no treatment is available.” Securing patient buy-in and commitment to surveillance efforts was viewed as a “herculean task…[and] a huge amount of time,” requiring largescale patient outreach and education.

### Inner setting

#### Relative priority and readiness

Even within the gastroenterology division where cirrhosis care takes place, focus group participants discussed the challenges of competing priorities: “even trying to engage [our] local GI department is a nightmare…colorectal screening priority is being measured, monitored, pushed and scrutinized so that’s their priority.” Participants explained that the catalyst for practice and process change would require making cirrhosis “a credible issue” and for it “to be a priority on everyone’s agenda.” Participants conveyed that readiness for implementation may not be uniform across all sites. There was an expectation that site size may affect organizational readiness, as one provider indicated “[we’re] not getting anything from our big sites, we were leaning on them to help us. [They] can’t expect the small sites to do it…they have to sail the ship.”

#### Leadership engagement and resources

Focus group participants identified that efforts should “come from top-down instead of us scrambling to the top.” Another participant cautioned that, “Patients need to buy in, but without leadership support, there will not be anything for the patients to buy in to.” Participants frequently reported needing increased resources to track patients, coordinate care, and conduct surveillances. One clinician hesitated that there was “more to do, [but] no one above us giving us time or resources to do it.” Participants spoke of demonstrating the need for HCC surveillance and increasing tension for change in leadership via building a compelling business case, “people [need to] understand the long-term complications and potential ways to save the hospital money with repeat hospitalizations if we have the support to manage the patients closely to try and prevent the re-hospitalizations.”

#### Structural characteristics and compatibility

Providers reported the structural characteristics of their sites impeded a multidisciplinary approach to cirrhosis care: “Structurally, if [we’re] going to screen more patients, each [site] will need to do more endoscopy, need more radiology availability, anesthesia support for endoscopy, ultrasound…[a] potential explosion of liver patients that is going to tax our system.” Many insisted the need to change clinic structure and existing workflows and systems to account for cirrhosis care because this “huge transformation” would require sites “dealing with totally different people and reestablishing relationships.”

#### Implementation facilitators

Facilitators participants described generally were in the domains of *implementation process* and *characteristic of individuals.*

#### Implementation process

##### Infrastructure and communication

Most participants reported positive experiences with the HIT collaborative for HCV which they believed would transfer to wider cirrhosis care improvement. They specifically commented on the collaborative structure and expanded network allowing for communication through more channels and elevating the priority of the clinical area. There was a universal appreciation for cross-region networking and team development as it made teams “not to operate inside your own little bubble, to get out and talk to people and share ideas.” Participants expected continued opportunities for collaboration (including peer-to-peer and expert-to-peer) via HIT virtual and in-person meetings, and training opportunities.

##### Data tools for reflecting and evaluating

Participants generally felt that having centralized and reliable data tools would facilitate quality improvement and help to “work smarter” and streamline care. The HIT had an established structure for monitoring and feeding back progress reports to regions for HCV, which was expected to continue. However, participants tempered their appreciation of the tools because they “don’t really have anybody dedicated to go through the dashboard or reach out to people.”

#### Characteristics of individuals

##### High self-efficacy due to prior success

Many participants underscored how their prior HIT experience prepared them for taking on new initiatives: “we have an idea of how to implement changes on a larger scale.” Gains from HCV work and the knowledge and skills fostered by the HIT contributed to heightened levels of confidence and self-efficacy that acted as facilitators for future cirrhosis care improvement: “It's been really exciting to see what VA has been able to accomplish when they put their minds to it.” Participants reported perseverance and comfort with change, and this served as a facilitator: “we lay the framework, we make sure everything is ready to roll and we do what we can until we get that support and then we go full throttle. We didn’t wait for the gate to be opened to start preparing to run.”

### Recommended strategies

The 10 barriers from the focus groups detailed above (i.e., adaptability, available resources, compatibility, complexity, external policy and incentives, leadership engagement, patient needs and resources, relative priority, readiness, and structural characteristics) were all entered into the CFIR-ERIC Matching Tool to generate a list of strategy recommendations. The left column of Table 1 lists the top 20 strategies recommended by the CFIR-ERIC Matching Tool based on those 10 barriers from the focus groups (order unimportant). For example, the barrier of relative priority was a focus group-identified barrier and is linked to two recommended ERIC strategies: “conduct local consensus discussions” and “conduct cyclical small tests of change.”

**Table 1:** Recommended strategies based on CFIR-ERIC mapping of top barriers

**Top 20 Recommended ERIC strategies**	**Actual strategy use**			**Strategy significance**
	**FY18**	**FY19**	**Top**	
1. Assess for readiness and identify barriers and facilitators	25%	13%		19
2. Conduct local consensus discussions	38%	23%	18	19
3. Promote adaptability	43%	42%	Both	Both
4. Conduct local needs assessment	24%	20%		19
5. Identify and prepare champions	44%	36%	Both	19
6. Build a coalition	40%	20%	18	19
7. Alter incentive/allowance structures	3%	3%		
8. Capture and share local knowledge	41%	26%	18	18
9. Tailor strategies	44%	40%	Both	Both
10. Conduct cyclical small tests of change	17%	16%		Both
11. Involve executive boards	19%	3%		
12. Involve patients and family members	25%	28%	19	19
13. Facilitation	14%	18%		
14. Develop a formal implementation blueprint	19%	12%		
15. Create a learning collaborative	30%	21%		18
16. Obtain and use patients and family feedback	11%	5%		18
17. Access new funding	24%	17%		
18. Inform local opinion leaders	30%	21%		18
19. Identify early adopters	14%	8%		
20. Fund and contract for clinical innovation	21%	15%		18

“Top” column denotes in which fiscal year the strategy was most frequently used. “Strategy significance” column denotes in which year the strategy was significantly associated with HCC surveillance

### Comparing recommended vs actual strategies

In Table 1, the percentages listed are the actual strategy use frequency in the first two years of implementation. The “top” column denotes in which year the strategy was a top-used strategy. Among the top 20 most recommended strategies (per the matching tool), six (30%) were actually used in FY18 and four (20%) were actually used in FY19. These included “build a coalition,” “capture and share local knowledge,” “conduct local consensus discussions,” “identify and prepare champions,” “involve patients and family members,” “promote adaptability,” and “tailor strategies.” On average, the remaining 53 strategies (i.e., 73 overall strategies minus 20 recommended) were not more or less frequently used in either year (FY18: 26% vs 25%; FY19: 19% vs 20%).

The “strategy significance” column in Table 1 indicates in which year the strategy was significantly positively associated with HCC surveillance. Overall, 35 of 73 (48%) of the actual strategies were effective in improving HCC performance in either FY18 and FY19 alone or in both years, compared to 14 of 20 (70%) of the top 20 recommended strategies. In the year after barriers were assessed (FY19), six strategies were associated with HCC surveillance including the five highest recommended strategies. This confirms the temporal sequence of barriers reported in FY18 being addressed in FY19.

In the first pivot year FY18, the recommended and effective strategies were: “inform local opinion leaders,” “participate in a collaborative,” “fund local efforts,” “capture and share local knowledge,” and “obtain and use patient feedback.” Strategies unique to the first year were related to barriers of opinion leaders, access to knowledge and information, and resources.

More planning and active implementation strategies were unique to the second year: “identify and prepare champions,” “build a coalition,” “conduct local needs assessments,” “assess for readiness,” “conduct local consensus discussions,” and “involve patients and family members.” Second-year strategies corresponded to barriers of champions, cosmopolitanism, readiness for implementation, relative priority, and patient needs and resources.

Of the recommended strategies, three were dynamic strategies used to optimize fit with context and improve outcomes and were effective in both years: “promote adaptability,” “tailor strategies,” and “conduct small tests of change.” Strategies effective in both years corresponded to barriers related to intervention characteristics of adaptability, trialability, and compatibility (from the inner setting but intervention-oriented).

### Actual strategies and expected barriers (reverse mapping)

The most popular actual strategies in each year were similar, with several notable differences. Five strategies were unique to FY18, six to FY19, and 14 overlapped between years totaling 25 strategies. Expected barriers were derived from these actual strategies using the CFIR-ERIC Matching Tool in reverse. Several strategies addressed the same barrier resulting in 15 unique expected barriers. Of these, seven (47%) had been previously identified through focus groups as an actual barrier (i.e., adaptability, available resources, compatibility, leadership engagement, patient needs and resources, relative priority, and structural characteristics).

Table 2 displays the most common strategies and their corresponding expected barriers and whether each was an actual barrier. Barriers addressed by the most popular strategies, but not explicitly identified during focus groups and included in the reverse matching tool query include access to knowledge and information, champions, cosmopolitanism, evidence strength and quality, executing, networks and communications, patient engagement, reflecting and evaluating, and self-efficacy. Note: Table 1 has original ERIC labels while Table 2 has cirrhosis-tailored ERIC labels and the FY18 strategy frequencies guide the order of strategies for FY19.

**Table 2:** Reverse mapping ERIC strategies to CFIR barriers

**Most used actual ERIC strategies (wording tailored to cirrhosis care)**	**Actual strategy use**		**Strategy significance**	**Expected CFIR barrier (per CFIR-ERIC Matching Tool)**	**Actual barrier**
	**FY18**	**FY19**			
• Use data warehousing techniques	73%	75%	Both	Reflecting & evaluating	
• Change physical structure and equipment	67%	50%		Available resources	a
• Change the record systems	60%	53%	19	Reflecting & evaluating	
• Use data experts to manage cirrhosis data	51%	37%	18	Reflecting & evaluating	
• Build on existing high-quality working relationships and networks to promote information sharing and problem-solving related to implementing cirrhosis care	49%	-	Both	Networks & communications	
• Facilitate the relay of clinical data to providers	49%	40%		Reflecting & evaluating	
• Tailor strategies to deliver cirrhosis care to address specific barriers in your center	44%	40%	Both	Compatibility	a
• Identify and prepare champions	44%	36%	19	Champions	
• Identify the ways cirrhosis care can be tailored to meet local needs and while still maintaining the core components of evidence-based care	43%	42%	Both	Adaptability	a
• Provide ongoing consultation with one or more cirrhosis treatment experts	43%	32%	Both	Self-efficacy	
• Distribute educational materials	43%	35%	18	Access to knowledge & information	
• Intentionally examine the efforts to promote cirrhosis care	43%	38%	18	Executing	
• Share the knowledge gained from quality improvement efforts with other sites outside your medical center	41%	-	18	Adaptability	a
• Conduct educational meetings	41%	44%		Access to knowledge & information	
• Build a local coalition/team to address challenges	40%	-	19	Cosmopolitanism	
• Develop reminder systems for clinicians	40%	36%	19	Leadership engagement	a
• Conduct local consensus discussions	38%	-	19	Relative priority	a
• Provide ongoing training in cirrhosis care	38%	33%	18	Self-efficacy	
• Provide clinical supervision around evidence-based cirrhosis care	37%	34%	18	Access to knowledge & information	
• Intervene with patients to promote uptake of and adherence to cirrhosis care	33%	-	Both	Patient engagement	
• Revise professional roles	-	35%		Structural characteristics	a
• Have an expert in cirrhosis care meet with providers to educate them	-	32%		Evidence strength & quality	
• Engage in efforts to prepare patients to be active participants in cirrhosis care	-	29%	Both	Patient engagement	
• Involve patients and family members	-	28%	19	Patient needs & resources	a
• Create new clinical teams	-	28%		Networks & communications	

Strategies are listed according to FY18 strategy frequencies; a dash indicates the strategy was not in the top 20 most frequently used in that particular year but was in the other year. “Strategy significance” column denotes in which year the strategy was significantly associated with HCC surveillance. “Actual barrier” column denotes whether the expected barrier was also an actual barrier identified as important by focus groups

The three most used strategies were consistently “use data warehousing techniques,” “change physical infrastructure,” and “change the record systems.” In turn, reverse mapping revealed these strategies were linked to expected CFIR barriers of reflecting and evaluating and available resources.

In the first year, sites did more “building on existing relationships,” “building a local coalition/team to address challenges,” “conducting local consensus discussions,” “sharing knowledge across sites,” and “intervening with patients to promote uptake of and adherence to cirrhosis care.” Unique first-year strategies corresponded to addressing expected barriers of networks and communication, cosmopolitanism, adaptability, relative priority, and patient engagement.

In the second year, sites did more “revising professional roles,” “creating new clinical teams,” “having cirrhosis care experts educate providers,” “providing ongoing training,” “involving patients and family members,” and “engaging in efforts to prepare patients to be active participants in cirrhosis care.” These strategies referred to barriers of evidence strength and quality, structural characteristics, networks and communication, patient needs and resources, and patient engagement.

## Discussion

Our mixed-methods evaluation illustrates overlap between recommended strategies and actual strategies used and shown to be effective, in the context of a national learning collaborative to improve cirrhosis care in the VA. For CFIR-based barriers identified through qualitative data collection with key stakeholders, strategies recommended by the CFIR-ERIC Matching Tool were more likely to be statistically positively associated with HCC surveillance across two years of implementation (70% actual vs 48% expected). Furthermore, the top-used strategies in the second year were positively associated with HCC surveillance, demonstrating a temporal relationship between barriers reported in the first year and strategy effectiveness in the second year. This work suggests the CFIR-ERIC Matching Tool’s recommendations can be useful in prescribing strategies to address barriers in a particular healthcare effort.

Our reverse application of the CFIR-ERIC Matching Tool based on the actual strategies used over two years found barriers shifted over time, speaking to the dynamic nature to fit the context and local needs. For example, the first pivot year barriers focused on opinion leaders, resources, and access to knowledge and information. In contrast, the second pivot year strategies concentrated on barriers of champions, cosmopolitanism, readiness, relative priority, and patient needs. The barriers consistent across years were less context-oriented and instead corresponded more to intervention qualities of adaptability, trialability, and compatibility. Strategy selection spanning multiple years must attend to the natural progression of context over time and the necessary strategy shifts [33]. Since barriers are rarely singular and momentary, further exploration of barrier combinations and the importance of their relative intensity is necessary to elucidate better strategy matching.

This study builds on the work of three unrelated studies that employed the CFIR-ERIC Matching Tool to identify strategies to mitigate recruitment and engagement to a cluster randomized trial in hospitals [34], implementation of infection control link nurses in acute care hospitals [35], and implementation of a patient-reported outcome measure portal in Dutch hospitals [36]. Across all three studies, the CFIR-ERIC Matching Tool recommended “identify and prepare champions” as the top strategy whereas “assesses for readiness and identify barriers and facilitators” was the leading recommended strategy in the current study. Among the top five strategies recommended by the CFIR-ERIC tool, the following strategies occurred in a least two out of three studies: “assess readiness and identify barriers and facilitators,” “conduct local consensus discussions,” and “inform local opinion leaders” [37]. This finding indicates that the tool has room to improve the specificity of matching. This is timely as the CFIR has been recently updated for the first time since its 2009 release [38].

Looking beyond the top five strategies across studies, the heterogeneity of recommendations is encouraging; however, each of these early adopter studies observed that while the tool was a helpful guide to matching strategies to barriers, there was a need to empirically evaluate the tool’s ability to recommend appropriate and effective strategies. This warrants not only a revision of the tool using updated empirical data such as presented here, but also more simplified and pragmatic methods for selecting strategies. However, several assumptions are underspecified in the current CFIR-ERIC Matching Tool and other emerging methods. For instance, Waltz et al. caution that a “single strategy may simultaneously address multiple barriers, depending on how it is operationalized” [6]. Similarly, strategies may work in concert to facilitate uptake and fidelity to cirrhosis care or other evidence-based guidelines. Application of the matching tool across settings using the same strategy nomenclature is critical, while continued real-world evaluation is needed to identify possible revisions and to ensure appropriate and replicable application.

### Limitations

While this evaluation identified actionable barriers during a pivot year, several limitations should be noted. First, these results must be interpreted in the context of VA and may not be generalizable to other settings. However, a third of experts contributing to CFIR-ERIC tool creation were employed or affiliated with the VA [6]. Second, there is the potential for social desirability bias when using focus groups and participants were at a regional level and may not reflect individual site determinants as well as perspectives of all local personnel. However, the focus groups conversely allowed the evaluation team to observe team dynamics and reach consensus. Another limitation of this study was that it was designed to capture an overarching impression of barriers and facilitators, rather than to disaggregate perceptions based on specialty or discipline. As such, we could not conduct true stratified analyses based on professional background. Focus group timing in the pivot year allowed for anticipated HCV and cirrhosis discussion reflected primarily in the implementation facilitators section. The translation of qualitative data into barriers for use in the CFIR-ERIC Matching Tool is a nascent method and should be interpreted with noted disclaimers. Finally, in the case of surveys, individual responses served as proxies for the site which were then compared to feedback received from across individuals during regional focus groups.

Additional limitations come from the use of the CFIR method itself. CFIR helps to translate diverse constructs from complementary theoretical models and frameworks informing an implementation evaluation, into a standardized set of factors. One limitation of determinant frameworks is that they are essentially checklists of possible factors rather than providing specific mechanisms. Another limitation of current best practices in identifying CFIR barriers is reducing rich qualitative data to a dichotomous presence or absence of each CFIR construct as a barrier. Consequently, another limitation is using discrete versus combinations of barriers. Not only are the individual construct nuances lost, but so too are their interrelationships. Despite these potential limitations, this evaluation uniquely assessed determinants of implementation both qualitatively and quantitatively and the results from each method converged on several themes that then informed policy.

## Conclusion

In conclusion, this national evaluation used an established implementation framework to understand factors related to HCC surveillance and cirrhosis care in VA. Leveraging facilitators and addressing barriers using implementation science and system redesign principles, VA has developed a roadmap to improve HCC surveillance and support clinicians through a national learning collaborative. Such efforts can serve as an example for other healthcare systems attempting to reduce the morbidity and mortality associated with complications of cirrhosis, most notably HCC. Future studies will examine how organizational factors inform strategy selection and identify associations of implementation strategies with clinical outcomes over the course of this multi-year collaborative.

## Data Availability

Data are available upon reasonable request from the corresponding author.

## Abbreviations

CFIR: Consolidated Framework for Implementation Research
ERIC: Expert Recommendations for Implementing Change
VA: Veterans Health Administration
QI: Quality improvement
HIT: Hepatic Innovation Team
HCV: Hepatitis C virus
HCC: Hepatocellular carcinoma
VISN: Veteran Integrated Service Network
APP: Advanced practice provider
